# *Caralluma europaea* (Guss.) N.E.Br.: Anti-Inflammatory, Antifungal, and Antibacterial Activities against Nosocomial Antibiotic-Resistant Microbes of Chemically Characterized Fractions

**DOI:** 10.3390/molecules26030636

**Published:** 2021-01-26

**Authors:** Fatima Ez-Zahra Amrati, Mohammed Bourhia, Hamza Saghrouchni, Meryem Slighoua, Andriy Grafov, Riaz Ullah, Essam Ezzeldin, Gamal A. Mostafa, Ahmed Bari, Samir Ibenmoussa, Dalila Bousta

**Affiliations:** 1 Laboratory of Biotechnology, Health, Agrofood and Environment (LBEAS), Faculty of Sciences Dhar El Mehraz, Sidi Mohamed Ben Abdellah University, Fez 30000, Morocco; slighoua.meryem@gmail.com (M.S.); boustadalila@gmail.com (D.B.); 2 Laboratory of Chemistry, Biochemistry, Nutrition, and Environment, Faculty of Medicine and Pharmacy, University Hassan II, Casablanca 20000, Morocco; ibenmoussa@yahoo.fr; 3 Department of Biotechnology, Institute of Natural and Applied Sciences, Çukurova University, Adana 01170, Turkey; hsaghrouchni@student.cu.edu.tr; 4 Department of Chemistry, Faculty of Sciences, Helsinki University, 00100 Helsinki, Finland; andriy.grafov@helsinki.fi; 5 Department of Pharmacognosy (MAPPRC), College of Pharmacy, King Saud University, P.O. Box 2457, Riyadh 11451, Saudi Arabia; 6 Department of Pharmaceutical Chemistry, College of Pharmacy, King Saud University, P.O. Box 2457, Riyadh 11451, Saudi Arabia; esali@ksu.edu.sa (E.E.); gmostafa@ksu.edu.sa (G.A.M.); abari@ksu.edu.sa (A.B.)

**Keywords:** *Caralluma europaea* (*Guss.*) N.E.Br., chemical characterization, anti-inflammatory, antibacterial, antifungal

## Abstract

*Caralluma europaea* (Guss.) N.E.Br.: (*C. europaea*) is a wild medicinal plant belonging to the family Apocynaceae. It is commonly used in traditional medicines for treating several diseases. The present work aims to evaluate the anti-inflammatory, antibacterial, and antifungal potentials of *C. europaea* fractions including hydro ethanol (ET CE), n-butanol (But CE), and polyphenol (Poly CE). The chemical composition of hydroethanol, n-butanol, and polyphenol-rich fractions from *C. europaea* were determined using GC-MS after silylation. The anti-inflammatory effect of hydroethanol, n-butanol, and polyphenol-rich fractions was studied by carrageenan-induced paw edema. Antibacterial and antifungal activities of hydroethanol, n-butanol, and polyphenol-rich fractions against Gram-positive bacteria, Gram-negative bacteria, and yeasts were assessed using the disc diffusion and micro-dilution assays. The findings of the chemical characterization affirmed the presence of interesting bioactive compounds in *C. europaea* fractions. The polyphenol-rich fraction was the best inhibitor of edema by75.68% after 6 h of treatment. The hydroethanol fraction was the most active against both bacteria and yeasts. This study contributes to society as it provides potential bioactive compounds in *C. europaea* extract, which may help in fighting nosocomial antibiotic-resistant microbes.

## 1. Introduction

For a long time, medicinal plants have been used to fight diseases. It is estimated that about 80% of the population based in developing countries use herbal medications for medication purposes [1]. Due to their accessibility and affordability, natural remedies are remarkably used in low-income settings for the treatment of several diseases, including inflammation, bacterial, and fungal infections [2].

Inflammatory diseases, bacterial and fungal infections are prevalent in developing countries due to socioeconomic and behavioral factors such as food hygiene, sanitation, and overcrowding effects [3]. Many studies reported that chronic inflammation and bacterial infections are associated with various diseases including cancer [4,5].

Chronic use of conventional drugs to fight inflammatory diseases, bacterial and fungal infections may have side effects, such as allergy, gastrointestinal disturbances, as well as drug resistance [6]. Thus, patients pay particular attention to modern drugs and look for alternative plant-based treatments. Moreover, the development of novel and effective natural-product-based plants to treat inflammation, bacterial, and fungal infections has received full attention and has become a considerable topic of several scientific communities [7].

Antimicrobial resistance is a complicated phenomenon where microbes developed resistance against commonly used antimicrobial drugs and antibiotics [8,9]. The tested bacteria in the present study such as *Staphylococcus aureus*, *Pseudomonas aeruginosa*, *Klebsiella pneumonia*, and *Escherichia coli pathogens* have been reported in previous works as multidrug-resistant, extensive drug-resistant, and even pan drug-resistant. In addition to the mentioned bacteria, *Candida albicans* is also recognized as one of the most common nosocomial agents, which is responsible for affecting more than 90% of patients with AIDS. The close association of *Candida* species infections and multidrug resistance is becoming belonging to the greatest growing health problem in medicines [10,11].

*Caralluma europaea* (*C. europaea*) is a leafless wild succulent species distributed in many Mediterranean countries including Morocco, Jordan, Egypt, Algeria, Tunisia, Libya, Spain, and Italy [12].

In traditional medicines, the aerial parts of *C. europaea* are recommended for being used as a juice, or as a powder mixed with honey or milk to treat inflammation, ulcer, diabetes, and bacterial infections [13]. Due to its presumed therapeutic effects, earlier literature reported some pharmacological activities of *C. europaea* such as antinociceptive, antiulcer, antihyperglycemic antioxidant, and cytotoxic activities [13,14,15].

Despite the wide use of *C. europaea* in traditional medicine, no well-developed research can provide scientific data on the potential activities of this species. Hence, the current study was conducted to study the chemical composition, anti-inflammatory, antibacterial, and antifungal activities of *C. europaea* extracts.

## 2. Results

### 2.1. GC–MS Analysis

**Figure 1:**
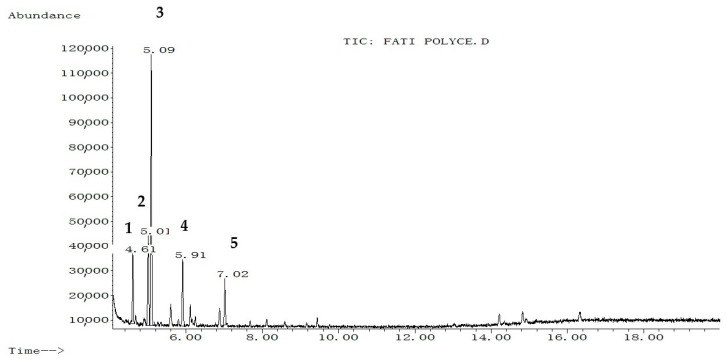
Chromatographic profile of hydroethanol fraction of *C. europaea* after silylation. **1**: Coumaran-3-one; **2**: 2-Phenylthiophene; **3**: Oxalic acid, **4**: Proline; **5**: Propanoic acid, 2-[(trimethylsilyl)oxy]-, trimethylsilyl ester.

**Figure 2:**
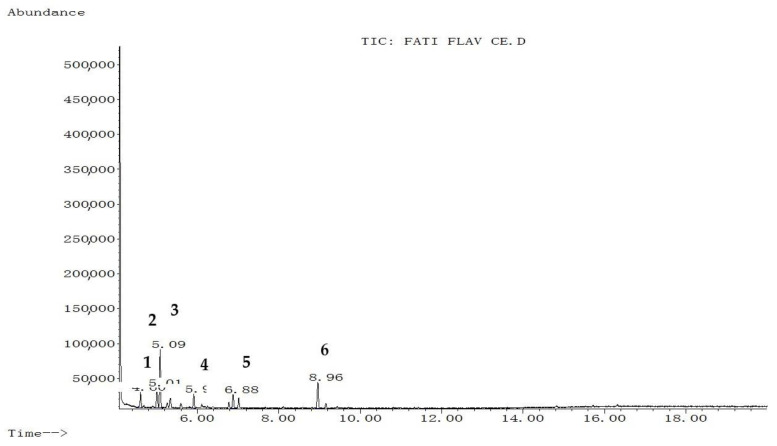
Chromatographic profile of the polyphenol-rich fraction of *C. europaea* after silylation. **1**: Benzoic acid, 4-methyl-2-trimethylsilyloxy-, trimethylsilyl ester; **2**: Phenol4-(3,4-dihydro-2,2,4-trimethyl-2H-1-benzopyran-4-yl); **3**: Cinnamic acid; **4**: Flavone, 2′-methoxy-; **5**: Butyric acid; **6***: p-coumaryl alcohol.*

**Figure 3:**
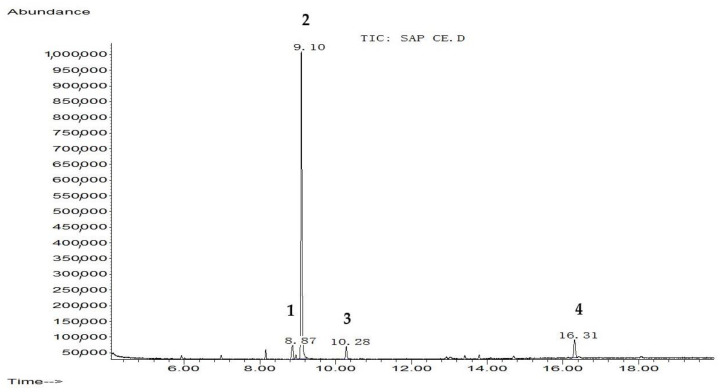
Chromatographic profile of n-butanol fraction of *C. europaea* after silylation. **1**: Benzoic acid, 4-methyl-2-trimethylsilyloxy-, trimethylsilyl ester; **2**: Elymoclavin; **3**: Benzenepropanoic acid, trimethylsilyl ester; **4**: 5-Hydroxy-6-methoxy-2-methyl-3-phenylbenzofuran (isoparvifuran).

The extraction yield of hydroethanol, butanol, and polyphenol-rich fractions were 13.25%, 12.9%, and 7.96% respectively. The chemical composition of each fraction from *C. europaea* (hydroethanol, *n*-butanol, and polyphenol) was determined by GC-MS after silylation. Results are summarized in Figure 1, Figure 2 and Figure 3, and Table 1, Table 2 and Table 3.

Silylation followed by GC-MS analysis of the hydroethanol fraction from *C. europaea* revealed the presence of several compounds including coumaran-3-one, 2-phenylthiophene, oxalic acid, proline, and propanoic acid, 2-[(trimethylsilyl)oxy]-, trimethylsilyl ester (Figure 1 and Table 1). Analysis of the polyphenol-rich fraction showed the presence of benzoic acid, 4-methyl-2-trimethylsilyloxy-, trimethylsilyl ester, phenol 4-(3,4-dihydro-2,2,4-trimethyl-2H-1-benzopyran-4-yl), cinnamic acid, flavone, 2′-methoxy-, butyric acid, and *p-coumaryl alcohol* (Figure 2 and Table 2). Analysis of the n-butanol fraction affirmed the presence of benzoic acid 4-methyl-2-trimethylsilyloxy, trimethylsilyl ester, elymoclavin, benzenepropanoic acid, trimethylsilyl ester, and 5-hydroxy-6-methoxy-2-methyl-3-phenylbenzofuran (isoparvifuran) (Figure 3 and Table 3).

### 2.2. Anti-Inflammatory Activity

The results of the anti-inflammatory effects of *C. europaea* fractions such as hydroethanol, n-butanol, and polyphenol-rich fractions at doses 100, 100, and 50 mg/Kg respectively, are summarized in Table 4.

The oral administration of hydroethanol, n-butanol, and polyphenol-rich fractions at doses 100, 100, and 50 mg/Kg induced significant anti-inflammatory effects when compared to the indomethacin drug (positive control). The animals treated with 100 mg/Kg of n-butanol fraction showed maximum inhibition of the paw edema, which reached 69.50%, 76.32%, and 76.70% after 4, 5, and 6 h of treatment respectively. No significant difference was observed between these results and indomethacin used as a standard drug at a dose of 10 mg/Kg (*p* > 0.05) (Table 4). The inhibited paw edema in mice treated with hydroethanolic (100 mg/Kg), and polyphenol (50 mg/kg) rich fractions reached 75.68% and 73.64%, respectively, after 3 h of the carrageenan intradermal injection.

### 2.3. Antibacterial Activity

The antibacterial activity of hydroethanol (ET CE), n-butanol (But CE), and polyphenol (Poly CE) rich fractions were evaluated using the disc diffusion method and micro-dilution assays. The results are presented in Table 5 and Table 6, as well as Supplementary material.

Table 5 represents the results of the minimum inhibitory concentration (MICs)obtained by using the micro-dilution method. All fractions showed almost equal effects with concentrations ranged from 6.25 to 12.5 mg/mL against Gram-negative bacteria. Regarding Gram-positive bacteria, the MIC of the Poly CE was 3.125 mg/mL against *Staphylococcus aureus*. The inhibition zone diameter bioassay showed that CE has a significant activity with an inhibition zone diameter of 12 mm against *Klebsiella pneumoniae* and *Staphylococcus aureus* (Table 6).

According to the results presented in Table 7, ET CE showed significant antifungal activity against *Candida albicans* and *Saccharomyces cerevisiae* with MIC of 6.25 mg/mL and 12.5 mg/mL respectively. Concerning the inhibition zone diameter assay (Table 8), But CE and ET CE induced an inhibition zone diameter against *Saccharomyces cerevisiae* and *Candida albicans* with 12 mm and 14 mm respectively.

## 3. Discussion

*C. europaea* is a medicinal plant historically used in traditional folk medicines to treat diseases including inflammation, bacterial, and fungal infections [15]. However, up to date, no more scientific data has been reported in the literature investigating the pharmacological activities of this plant. As consequence, the currents work was undertaken to study antibacterial, antifungal, and anti-inflammatory activities of *C. europaea* growing in Morocco.

In the last decades, the prevalence of inflammatory diseases has increased throughout the world. Steroidal and non-steroidal anti-inflammatory drugs are mostly used in treating these diseases. However, their use for a long time may have serious side effects that can be mortal. This can explain the interest of many people in herbal remedies as a potential alternative treatment for inflammatory diseases [16]. The inflammatory process is a complex biological response of the body including the overproduction of cytokines or pro-inflammatory molecules through the activation of different signaling pathways.

The findings obtained in the present work showed that hydroethanol, *n*-butanol, and polyphenol-rich fractions from *C. europaeas* showed an important anti-inflammatory effect. The observed inflammatory effect can be due to benzoic acid and isoparvifuran compounds identified in the *n*-butanol fraction chemically characterized by GC-MS (Figure 3 and Table 3). Benzoic acid derivatives were found to have a potent effect to inhibit cyclo-oxygenase activity. Consequently, these chemicals can act as anti-inflammatory agents and can be used to treat various inflammatory disorders [17,18]. Isoparvifuran and flavones compounds displayed potent anti-inflammatory activity as reported elsewhere [19,20,21].

The anti-inflammatory activity of *C. europaea* polyphenol-rich fraction might be also related to phytochemical compounds identified by HPLC such as ferulic acid, quercetin, myricetin, gallic acid, and hesperetin, as reported in the earlier work [15]. Many literature studies reported that ferulic acid, quercetin, myricetin, gallic acid, and hesperetin were found to possess potent anti-inflammatory properties that act through different biological pathways [5,22,23,24,25]. These findings are in agreement with many literature studies reporting that ethanolic extracts from *Caralluma arabica* and *Caralluma attenuate* as close species to our studied plant exhibited significant anti-inflammatory effects via their chemically identified compounds such as luteolin-4%-*O*-neohesperidoside,lutolin-4%-*O*-[a-(l-rhamnopyranosyl-(12)-b-d-glucopyranoside)] [26,27]. Propanoic acid identified by GC-MS in the hydroethanol fraction of *C. europaea* as well as cinnamic acid can be also responsible for the observed anti-inflammatory effects (Figure 1 and Table 1) (Figure 2 and Table 2) [28,29,30].

Regarding antibacterial activities investigated in the present work, almost all fractions from *C. europaea* were more active on Gram-positive bacteria and yeasts. Our results showed that Gram-positive bacteria were generally more sensitive to plant fractions than Gram-negative bacteria. These results were closely similar to some studies showing the sensitivity of Gram bacteria to plant extracts more than Gram-negative [31,32]. The structure of the cell envelope may be responsible for this difference since Gram-negative bacteria have additional periplasmic space and a rigid layer between the outer and cytoplasmic membrane, which limits the diffusion of hydrophobic compounds [33]. Our results showed that *P. aeruginosa* (Gram-negative) was the most resistant bacteria to plant fractions. This result consistent with the previous literature, which showed the resistance of *P. aeruginosa* to conventional antimicrobials [34,35,36].

The antibacterial effect of our fractions can be attributed to the effect of benzoic acid (Figure 3 and Table 3) since earlier work reported the inhibitory effect of benzoic acid on the proliferation of bacteria and yeasts [37]. Proline-rich antimicrobial peptides (PR-AMPs) (Figure 1 and Table 1) characterized by a high content of proline residues can play a role in inhibiting several pathogenic bacteria and yeasts [38]. Cinnamic acid (Figure 2 and Table 2) was found to have antibacterial activity by disrupting bacteria membranes [39]. Benzenepropanoic acid (Figure 3 and Table 3,) was also reported to be effective against different bacteria such as *Escherichia coli*, *Klebsiella pneumonia*, *Pseudomonas aeruginosa,* and some fungi including *candida albicans* [40]. Moreover, carboxylic acids, including oxalic acid and propanoic acid identified by GC-MS analysis in the hydroethanol fraction of *C. europaea* (Figure 1 and Table 1), might be involved in antifungal activity [41,42]. Moreover, the hydrophobicity of these compounds facilitates their penetration between the lipid components of bacterial membrane and mitochondria, which increases the membrane permeability and leads to the eventual death of bacteria [43,44]. This mechanism of action could be involved in the antibacterial effect induced by our plant fractions. Furthermore, the antimicrobial effect can result from individual compounds or synergy between a lot [45]. The findings found in this work are in agreement with the previous literature, which showed that essential oils from *C. europaea* exhibited antibacterial activities against Gram-positive bacteria such as *K. pneumonia* and *P. aeruginosa*. Potential antifungal effects of *Caralluma. europaea* essential oil might also be reported elsewhere [46].

The antimicrobial effect shown by this plant can be attributed to the presence of monoterpene hydrocarbons, including α- terpinene, α-pinene, and β-pinene, and oxygenated monoterpenes. These compounds showed antimicrobial activity by inducing cell integrity perturbation, inhibition of cell respiration, as well as increasing membrane permeability [31,44,47].

## 4. Materials and Methods

### 4.1. Solvents and Reagents

Dichloromethane, ethanol, methanol, chloroform, ethyl acetate, n-butanol, n-hexane, and N-Trimethylsilyl-N-methyl trifluoroacetamide were purchased from Sigma Aldrich (Munich, Germany).

### 4.2. Plant Material

*C. europaea* was collected from the Imouzzer region at the Middle Atlas Mountains, Morocco in April 2018. The plant was authenticated by the botanist Bari Amina, and given the voucher specimen#18I4C001 before being deposited at the herbarium of the Department of Biology, Faculty of Sciences Dhar El Mahraz, Sidi Mohammed BenAbdallah Fes University, Morocco. Afterward, the aerial parts were dried in the shade in a well-ventilated room for one week before being ground into powder using a blender.

### 4.3. Animal Material

Weighing between 170 and 246 g, male and female adult Wistar rats (ethic approval number: 04/2019/LBEAS) turn 8 weeks of age were used to perform this work. Animals were obtained from the animal house of the Department of Biology, Faculty of Sciences Dhar El Mahraz, Sidi Mohammed Ben Abdallah Fes University, Morocco. Animals were housed under controlled laboratory conditions with a temperature of 23 ± 2 °C and 12 h light/dark cycle. They were also allowed free access to food and water ad libitum. The Animal Ethics Review Committee at the faculty of Sciences, Fez University Morocco, reviewed and approved this study. The use of laboratory animals in all experimental procedures was conducted according to the ethical guidelines for the care and the use of laboratory animals [48].

### 4.4. Extraction of Hydroethanol, n-Butanol, and Polyphenol-Rich Fractions from C. europaea

#### 4.4.1. Preparation of Hydroethanol and *n*-Butanol Fractions

A total of 10 g of powder of *C. europaea* was extracted with 100 mL of hydroalcoholic solution (7: 3 *v*/*v* Alcohol: distilled water) for 15 min at 25 °C using the sonicator (Model GT Sonic). The mixture was filtered before being concentrated at 40 °C under vacuum using a rotary evaporator (model BÜCHI 461) [15]. The residue was then stored at 4 °C until further use.

#### 4.4.2. Preparation of the Polyphenol-Rich Fraction

The dried powder of *C. europaea* aerial parts (10 g) was extracted three times with 30 mL of methanol. Thereafter, the mixture was concentrated under reduced pressure and low temperature. The dry extract obtained was dissolved in 50 mL of water and successively extracted three times again with 20 mL of hexane, chloroform, and ethyl acetate. Afterward, the ethyl acetate layer was evaporated at 40 °C under vacuum using a rotary evaporator (Model BÜCHI 461). The residue was redissolved in 30 mL of water and freeze-dried to obtain the polyphenol extract [49]. The residue was then stored at 4 °C until further use.

### 4.5. GC-MS Analysis

One milligram of each fraction (hydroethanol, *n*-butanol, and polyphenol-rich fraction) was extracted with 0.5 mL of dichloromethane. Afterward, 200 µL of *N*-Trimethylsilyl-*N*-methyl trifluoroacetamide (MSTFA) was added to the mixture before being incubated at 37 °C for 30 min. 0.1 μL of each final extract was injected into the GC-MS apparatus equipped with a capillary column model number (Agilent 19091S-433; Diameter: 0.25 mm, Length: 30 m, Film thickness: 0.25 µm) [50]. The oven temperature program was set to 60–300 °C for 10 min and then maintained at 300 °C for 20 min. The injector temperature was set to 260 °C, and the detector temperature to 250 °C. Helium was used as a carrier gas with a total flow of 31.4 mL/min and the split ratio was set to 30:1. The identification of the silylated compounds was conducted by comparing the retention times with those of the standards obtained from the database of the GC-MS Wiley 7n.l.

### 4.6. Anti-Inflammatory Activity

The anti-inflammatory effect of each studied fraction (hydroethanol, n-butanol, and polyphenol) was evaluated by carrageenan-induced paw edema according to the method described in the literature [51,52]. Animals were divided into five groups with 5 in each group and then treated as follows:

Group 1: negative control (0.9% NaCl)
Group 2: positive control (10 mg/Kg of indomethacin)
Group 3: hydroethanol fraction (100 mg/Kg)
Group 4: polyphenol-rich fraction (50 mg/Kg)
Group 5: n-butanol fraction (100 mg/Kg)

After one hour of gastric gavage, the inflammation was induced by injecting 0.1 mL of the carrageenan agent in 0.9% saline solution into the right-hand paw of rats. The initial paw size was taken before the injection of carrageenan and after 3, 4, 5, and 6 h of treatment. The average increase in the paw size of each group was determined and compared to both the positive and the negative control. The percentage of inhibition of edema was calculated as follows:

% inhibition = ((St-S0) control − (St-So) treated/(St-S0) control) × 100

where is the paw size after the carrageenan injection and S0 is the initial paw size before the carrageenan injection.

### 4.7. Antibacterial Activity

#### 4.7.1. Growing Media

Muller Hinton Agar MHA medium was used for bacteria growth, and the Sabouraud SB medium was used for yeast growth. Micro-dilution technique for both yeast and bacteria was done using Sabouraud SBB and Muller Hinton MHB respectively. All media were autoclaved at 120 °C for 20 min before the use [53].

#### 4.7.2. Bacterial and Yeast Strains

The antimicrobial activity of the studied fractions was carried out using Gram-positive bacteria; *Staphylococcus aureus*, and Gram-negative bacteria; *Escherichia coli* (ATB: 57) B6N, *Pseudomonas aeruginosa*, and *Klebsiella pneumonia*. The antifungal activity of the studied fractions was studied using *Candida albicans* ATCC10231 and *Saccharomyces cerevisiae* ATCC9763.

#### 4.7.3. Inoculum Standardization

The microbial inoculum was performed by taking 3 colonies from the fresh culture (24-h), which were aseptically collected and suspended in 0.9% sterile saline solution with a density of 0.5 McFarland [54]. The bacterial suspensions were adjusted to have approximately 1–2 × 10^8^ CFU/mL, and the yeast suspensions 1–5 × 10^6^ CFU/mL. The absorbance of the solution was read by a UV-Visible spectrophotometer (Agilent technologies, Munich, Germany) (Selecta, E. U) at λ = 625 nm [55].

#### 4.7.4. Disc Diffusion Method

The plates containing the agar medium MH and YPG were inoculated with 1 mL of bacterial and fungal suspensions respectively before being dried for 10 min. Afterward, 6 mm sterile discs were impregnated with 10 μL of each tested fraction (ET CE, But CE, Poly CE). The antibiogram discs; 1.67 mg/disc of ampicillin (AMP), 0.02 mg/disc of streptomycin (STR), and the antifungal disc of 5 mg/disc of fluconazole (FLU) were used as standard drugs. Finally, the plates inoculated with bacteria were incubated again at 37 °C and those inoculated with yeasts at 30 °C for 24 h. The growth inhibition zones were determined in mm [55,56].

#### 4.7.5. Determination of the Minimum Inhibitory Concentration (MIC)

The MIC (the minimum concentration that inhibits microbial growth) was performed using a micro-dilution assay in 96-well plates [53]. First, the ET CE, But CE, Poly CE, AMP, STR, FLU, and the microbial suspensions in 0.5 McFarland were diluted in the broth culture medium. Afterward, 50 μL of the culture medium was deposited into a microplate well previously filled with 100 μL of the test fraction except wells dedicated to growth control (positive growth control). Next, micro-dilutions were made by transferring 50 μL from the first well to the second successively (½ dilution factor ½). The inoculation was carried out by putting 50 μL into wells of the microbial suspension whose turbidity was adjusted to 0.5 McFarland and then were diluted in the broth culture medium. Subsequently, the microplate was incubated under agitation for 24 h at 37 °C for the bacteria and 30 °C for the yeasts. Then, twenty microliters of 2,3,5-triphenyl tetrazolium chloride (TTC), BIOKAR company mixed with an aqueous solution (1%) was added to all wells for reading. Finally, a pinkish coloration appeared when there is growth after incubation for 2 h. MIC was defined as the lowest concentration that does not produce a pink color, while the well without bacterial growth remained colorless [17,18].

### 4.8. Statistical Analysis

The results obtained were expressed as means ± SEM (standard error of the mean). Data were statistically analyzed using one-way analysis of variance (ANOVA) and student’s *t*-test to perform the comparison using GraphPad Prism 7. *p*-value < 0.05 was considered significant.

## 5. Conclusions

The chemical study of hydroethanol, *n*-butanol, and polyphenol-rich fractions from *Caralluma europaea* aerial parts revealed the presence of many potentially active compounds that are involved in antibacterial, antifungal, and anti-inflammatory activities shown in this research work. This study can serve patient wellbeing as it provides potentially bioactive compounds contained in *C. europaea*, which can be used as alternative agents to fight inflammatory diseases and nosocomial antibiotic-resistant microbes.

## Figures and Tables

**Table 1:** GC-MS identified compounds in the hydroethanol fraction of *C. europaea*.

Rank Peak No	Name of Compound	Molecular Weight (g/mol)	Structural Formula	% Area	RT (min)
1	Coumaran-3-one	134.13	C_8_H_6_O_2_	12.388	4.61
2	2-Phenylthiophene	160	C_10_H_8_S	17.615	5.01
3	Oxalic acid	90.03	C_2_H_2_O_4_	49.122	5.09
4	Proline	115.13	C_5_H_9_NO_2_	12.718	5.91
5	Propanoic acid, 2-[(trimethylsilyl)oxy]-, trimethylsilyl ester	234	C_9_H_22_O_3_Si_2_	8.157	7.02

**Table 2:** GC-MS identified compounds in the polyphenol-rich fraction of *C. europaea*.

Rank Peak No	Name of Compound	Molecular Weight (g/mol)	Structural Formula	% Area	RT (min)
1	Benzoic acid, 4-methyl-2-trimethylsilyloxy-, trimethylsilyl ester	296	C_14_H_24_O_3_Si_2_	9.610	4.60
2	Phenol4-(3,4-dihydro-2,2,4-trimethyl-2H-1-benzopyran-4-yl)	268	C_18_H_20_O_2_	14.013	5.01
3	Cinnamic acid	148.16	C_9_H_8_O_2_	37.271	5.09
4	Flavone, 2′-methoxy-	252	C_16_H_12_O_3_	9.297	5.9
5	Butyric acid	88.11	C_4_H_8_O_2_	9.859	6.88
6	*p*-coumaryl alcohol	150.17	C_9_H_10_O_2_	19.950	8.96

**Table 3:** GC-MS identified compounds in the n-butanol fraction of *C. europaea*.

Rank Peak No	Name of Compound	Molecular Weight (g/mol)	Structural Formula	% Area	RT (min)
1	Benzoic acid, 4-methyl-2-trimethylsilyloxy-, trimethylsilyl ester	296	C_14_H_24_O_3_Si_2_	5.536	8.87
2	Elymoclavin	254.33	C_16_H_18_N_2_O	83.829	9.10
3	Benzenepropanoic acid, trimethylsilyl ester	222	C_12_H_18_O_2_Si	3.758	10.28
4	5-Hydroxy-6-methoxy-2-methyl-3-phenylbenzofuran (isoparvifuran)	254	C_16_H_14_O_3_	6.877	16.31

**Table 4:** Anti-inflammatory effects of *C. europaea* fractions on carrageenan-induced paw edema.

Treatment Groups	Basal Diameter (cm)	Paw Size after the Carrageenan Injection (Mean ± SEM)/Percent Inhibition of Edema			
		3 Hour	4 Hour	5 Hour	6 Hour
NaCl	2.3 ± 0.01581	2.6 ± 0.04472	2.8 ± 0.0547	2.68 ± 0.0158	2.520 ± 0.0209
Indomethacin^®^ 10 mg/kg	2.220 ± 0.03742	2.420 ± 0.03742 ** 33.33%	2.362 ± 0.0348 * 71.60%	2.300 ± 0.0273 78.95%	2.266 ± 0.0331 79.09%
ET CE 100 mg/kg	2.340 ± 0.02449	2.556 ± 0.0250 *** 28%	2.534 ± 0.0271 *** 61.20%	2.474 ± 0.0208 ** 64.74%	2.398 ± 0.0217 73.64%
Poly CE 50 mg/kg	2.315 ± 0.00866	2.523 ± 0.0062 *** 30.38%	2.473 ± 0.0062 *** 68.5%	2.410 ± 0.0070 *** 75%	2.369 ± 0.0060 *** 75.68%
But CE 100 mg/kg	2.350 ± 0.02887	2.555 ± 0.0295 *** 31.67%	2.503 ± 0.0246 ** 69.50%	2.440 ± 0.0291 76.32%	2.401 ± 0.0282 76.70%

Values are expressed as means ± SEM. (*n* = 5), *p* < 0.05 considered statistically significant compared to the control and reference drug (indomethacin 10 mg/mL). *: Significant, **: high significant; ***: extremely significant.

**Table 5:** MCI results of *C. europaea* fractions against bacterial species in (mg/mL).

Fractions	Gram-Negative Bacteria			Gram-Positive Bacteria
	*Escherichia coli*	*Klebsiella pneumoniae*	*Pseudomonas aeruginosa*	*Staphylococcus aureus*
But CE	6.25	25	6.25	6.25
Poly CE	6.25	12.5	12.5	3.125
ET CE	12.5	12.5	25	12.5
STR	0.25	0.003	Resistant	0.062
AMP	Resistant	Resistant	Resistant	Resistant

**Table 6:** Inhibition zone diameter of *C. europaea* fractions against bacterial species in (mm).

Fractions	Gram-Negative Bacteria			Gram-Positive Bacteria
	*Escherichia coli*	*Klebsiella pneumoniae*	*Pseudomonas aeruginosa*	*Staphylococcus aureus*
But CE	10	7	Resistant	9
Poly CE	Resistant	Resistant	Resistant	8
ET CE	9	12	10	12
Streptomycine	Resistant	Resistant	Resistant	9
Ampicilline	Resistant	Resistant	Resistant	Resistant

**Table 7:** MCI of *C. europaea* fractions against yeasts in (mg/mL).

Fractions	*Candida albicans*	*Saccharomyce sereveseae*
But CE	25	25
Poly CE	50	50
ET CE	6.25	12.5
Fluconazole	0.4	0.2

**Table 8:** Inhibition diameter of *C. europaea* fractions against yeasts in (mm).

Fractions	*Candida albicans*	*Saccharomyce sereveseae*
But CE	Resistant	14
Poly CE	Resistant	Resistant
ET CE	12	Resistant
Fluconazole	21	27

## Data Availability

The data used to support the findings of this study are available from the corresponding author upon request.

## Supplementary Materials

The following are available online. Figure S4: Determination of minimum inhibitory concentrations (MIC) of hydroethanol, butanol, and the polyphenol-rich fraction of *Caralluma europaea* against Pseudomonas aeruginosa, Escherichia coli, Klebsiella pneumonia, Staphylococcus aureus, Saccharomyces cerevisiae, and Candida albicans.

## Abbreviations

CE: *Carallumaeuropaea* (Guss.) *N.E.Br.*; *C. europaea*: *Caralluma europaea*; ET CE: Hydro-ethanol fraction from *Carallumaeuropaea*; But CE: n-butanol fraction from *Carallumaeuropaea*; Poly CE: polyphenol-rich fraction from *Carallumaeuropaea*; AMP: Ampicillin; STR: Streptomycin; FLU: Fluconazole; MSTFA: Trimethylsilyl-N-methyl trifluoroacetamide; *K. pneumonia*: Klebsiella pneumonia; *P. aeruginosa*: Pseudomonas aeruginosa; MCI: Minimal Inhibitory Concentration; MeOH: Methanol; GC-MS: Gas Chromatography/Mass Spectrometry; MHA: Muller Hinton Agar; SB: Sabouraud; SBB: Sabouraud Broth; MHB: Muller Hinton.
