# Supplementary material for: Caralluma europaea (Guss.) N.E.Br.: Anti-Inflammatory, Antifungal, and Antibacterial Activities against Nosocomial Antibiotic-Resistant Microbes of Chemically Characterized Fractions
Source: Molecules. 2021 Jan 26;26(3):636. doi: 10.3390/molecules26030636 (PMC7865290; doi:10.3390/molecules26030636)
Supplement: Supplementary file 1 [file molecules-26-00636-s001.pdf]

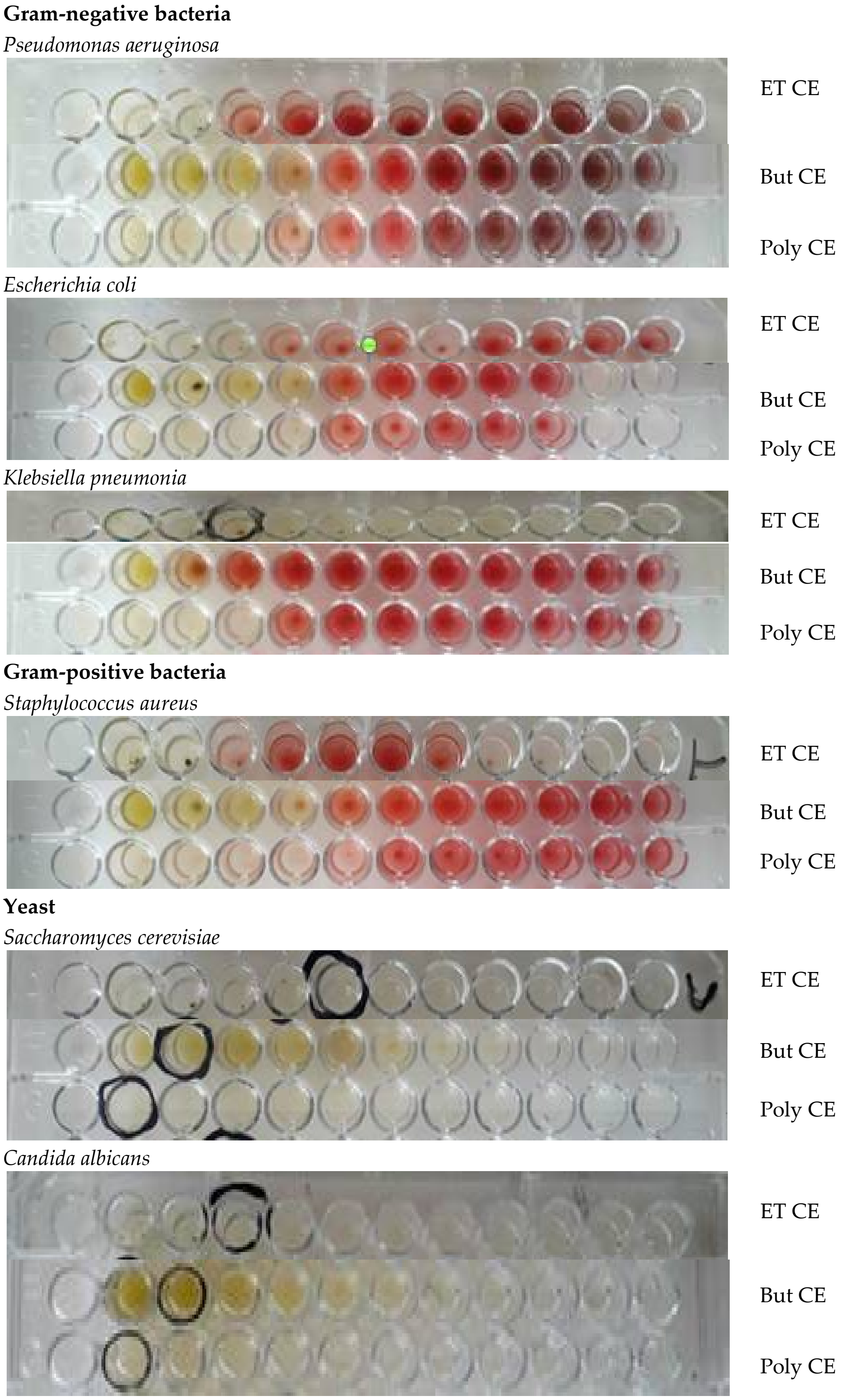

Figure 4. Determination of minimum inhibitory concentrations (MIC) of hydroethanol, butanol, and the polyphenol-rich fraction of *Caralluma europaea* against *Pseudomonas aeruginosa, Escherichia coli, Klebsiella pneumonia, Staphylococcus aureus, Saccharomyces cerevisiae*, and *Candida albicans*.
